# Supplementary material for: scMINER: a mutual information-based framework for clustering and hidden driver inference from single-cell transcriptomics data
Source: Nat Commun. 2025 May 8;16:4305. doi: 10.1038/s41467-025-59620-6 (PMC12062461; doi:10.1038/s41467-025-59620-6)
Supplement: Supplementary file 1 — Supplementary Information [file 41467_2025_59620_MOESM1_ESM.pdf]

Supplementary Information

scMINER: a mutual information-based framework for clustering and hidden driver inference from single-cell transcriptomics data

Pan, et al.

This document contains:

Supplementary Figures 1-13

Supplementary Tables 1-3

Supplementary References

Supplementary Figures

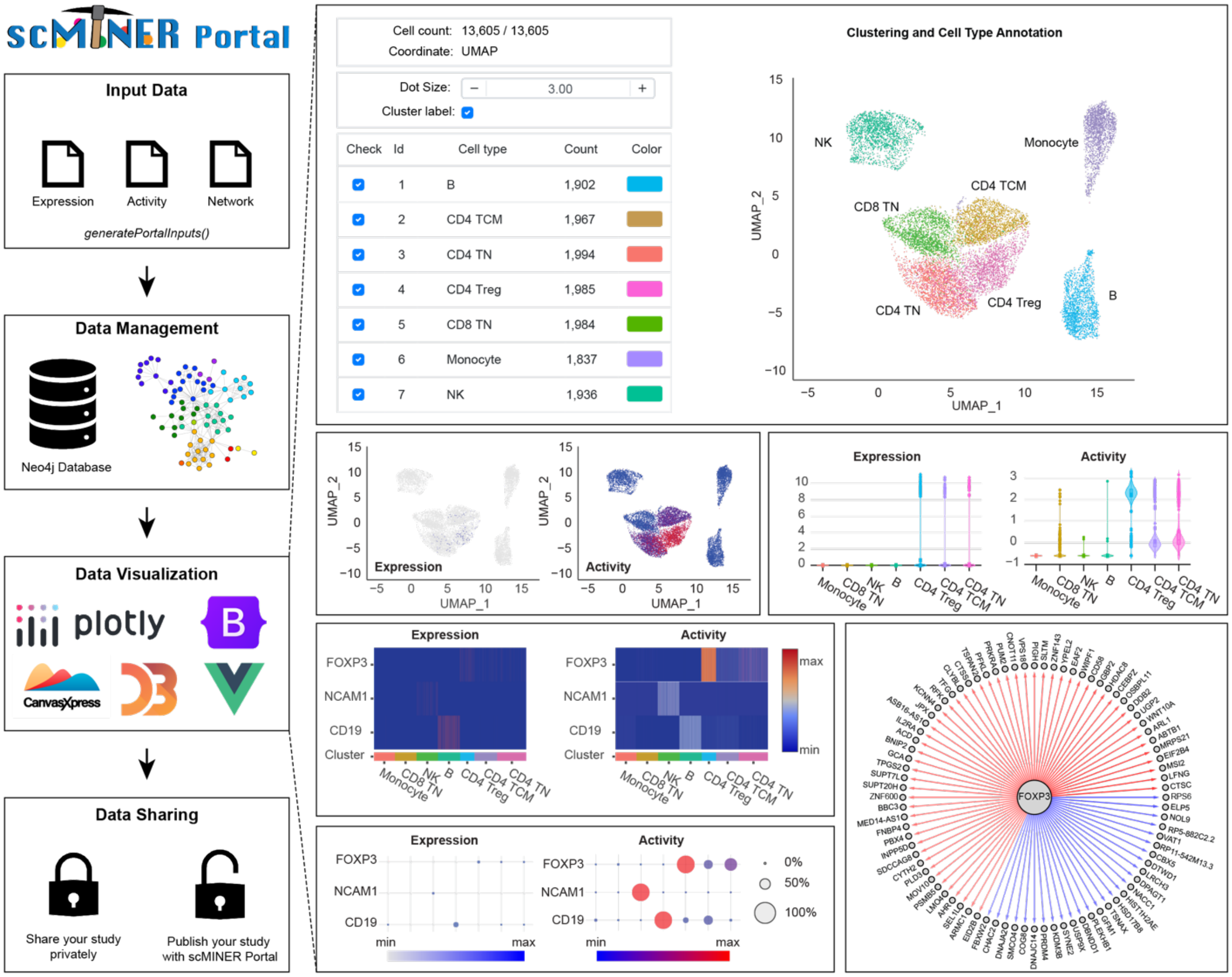

Supplementary Figure 1 | Overview of scMINER Portal. As a component of scMINER framework, scMINER Portal is a web-based data platform designed for visualizing, exploring and sharing the scRNA-seq data analysis conducted by scMINER. On the back end, scMINER Portal takes the standard outputs of scMINER analysis, including normalized gene expression matrix, activity matrix and TF and SIG networks, as the inputs and coverts them into a graph using the Neo4j database system. On the front end, scMINER Portal integrates multiple charting library to provide comprehensive, dynamic and interactive data visualizations for each single cell study. In addition to data visualization, scMINER Portal also provides an accessible way for researchers to share their studies privately or publicly, and explore other single cell datasets that already publicly available.

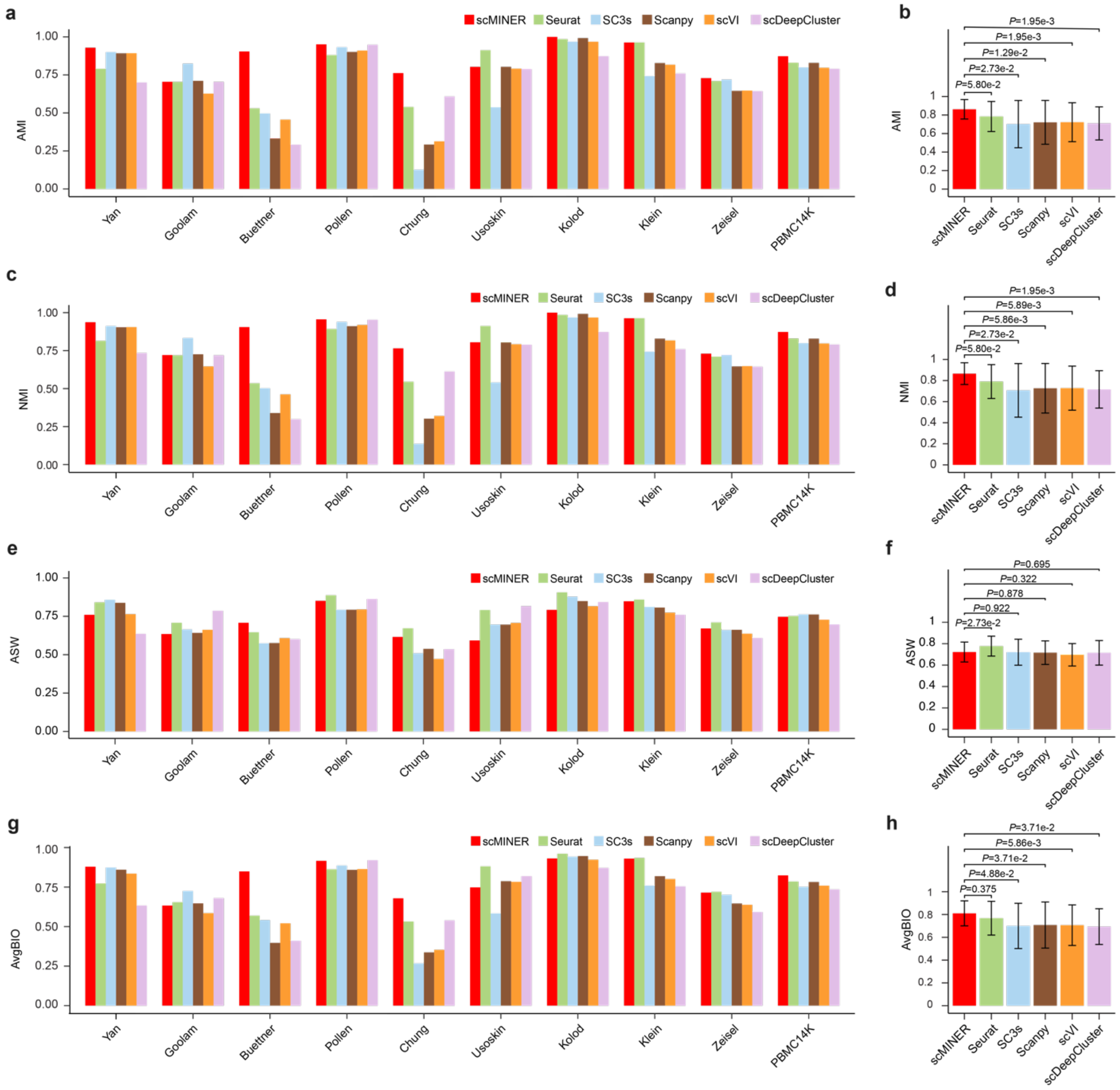

Supplementary Figure 2 | Benchmarking of scMINER for cell clustering accuracy using alternative clustering metrics. a-h, Bar plots indicating the accuracy of clustering measured by AMI (Adjusted Mutual Information, **a,b**), NMI (Normalized Mutual Information, **c,d**), ASW (Average Silhouette Width, **e,f**) and AvgBIO (Average BIO score, **g,h**) of scMINER and other five benchmarked algorithms across 10 ground truth datasets. Each bar in **b,d,f,h** represents the average AMI, NMI, ASW, AvgBIO of each method across all datasets, and error bars represent the standard deviation. *P* value was estimated using paired samples Wilcoxon test, two-tailed. AMI: Adjusted Mutual Information; NMI: Normalized Mutual Information; ASW: Average Silhouette

Width; AvgBIO: Average Biological conservation score. Source data are provided as a Source Data file.

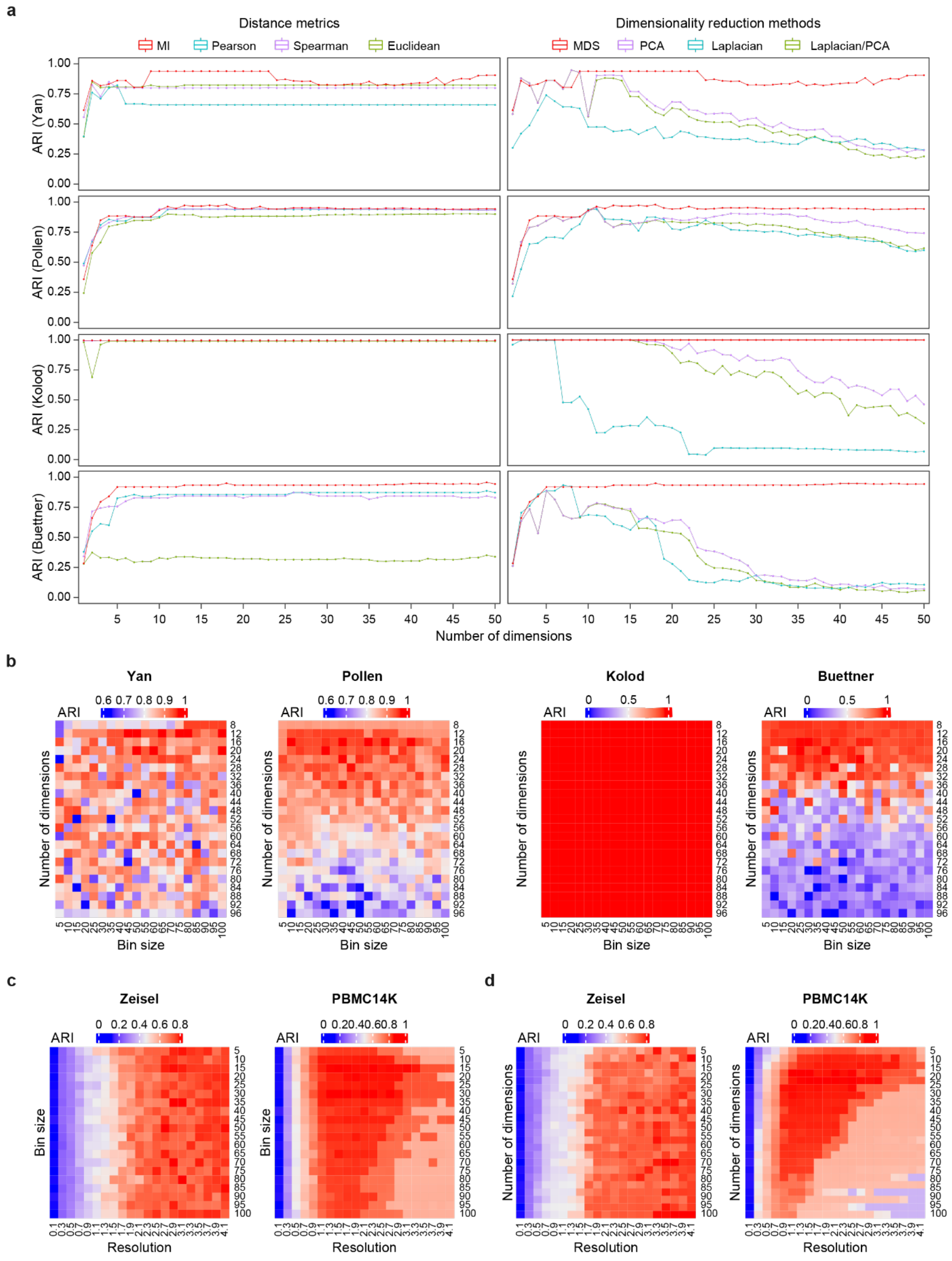
a
Distance metrics
MI
Pearson
Spearman
Euclidean
Dimensionality reduction methods
MDS
PCA
Laplacian
Laplacian/PCA
ARI (Yan)
ARI (Pollen)
ARI (Kolod)
ARI (Buettner)
Number of dimensions
b
Yan
Pollen
Kolod
Buettner
ARI
Number of dimensions
Bin size
c
Zeisel
PBMC14K
Bin size
Resolution
d
Zeisel
PBMC14K
Number of dimensions
Resolution

Supplementary Figure 3 | Benchmarking of scMINER for cell clustering robustness across varying dimensionalities and bin sizes. a, Line plots showing the impact of the number of dimensions on clustering accuracy for mutual information and other three cell-cell distance metrics (left panels), as well as MDS and other dimensionality reduction methods (right panels) across four ground-truth datasets of gold-standard, Yan, Pollen, Kolod, and Buettner. The dots on each line indicate the ARI (Adjusted Rand Index) values. MDS: multidimensional scaling. **b**, Heatmaps indicating the impact of the number of dimensions and bin size on clustering accuracy of MICA-MDS mode across the four ground-truth datasets of gold-standard. **c,d**, Heatmaps showing the impact of bin size (**c**) and number of dimensions (**d**) on clustering accuracy of MICA-GE mode at varying Louvain resolutions across Zeisel and PBMC14K datasets. Source data are provided as a Source Data file.

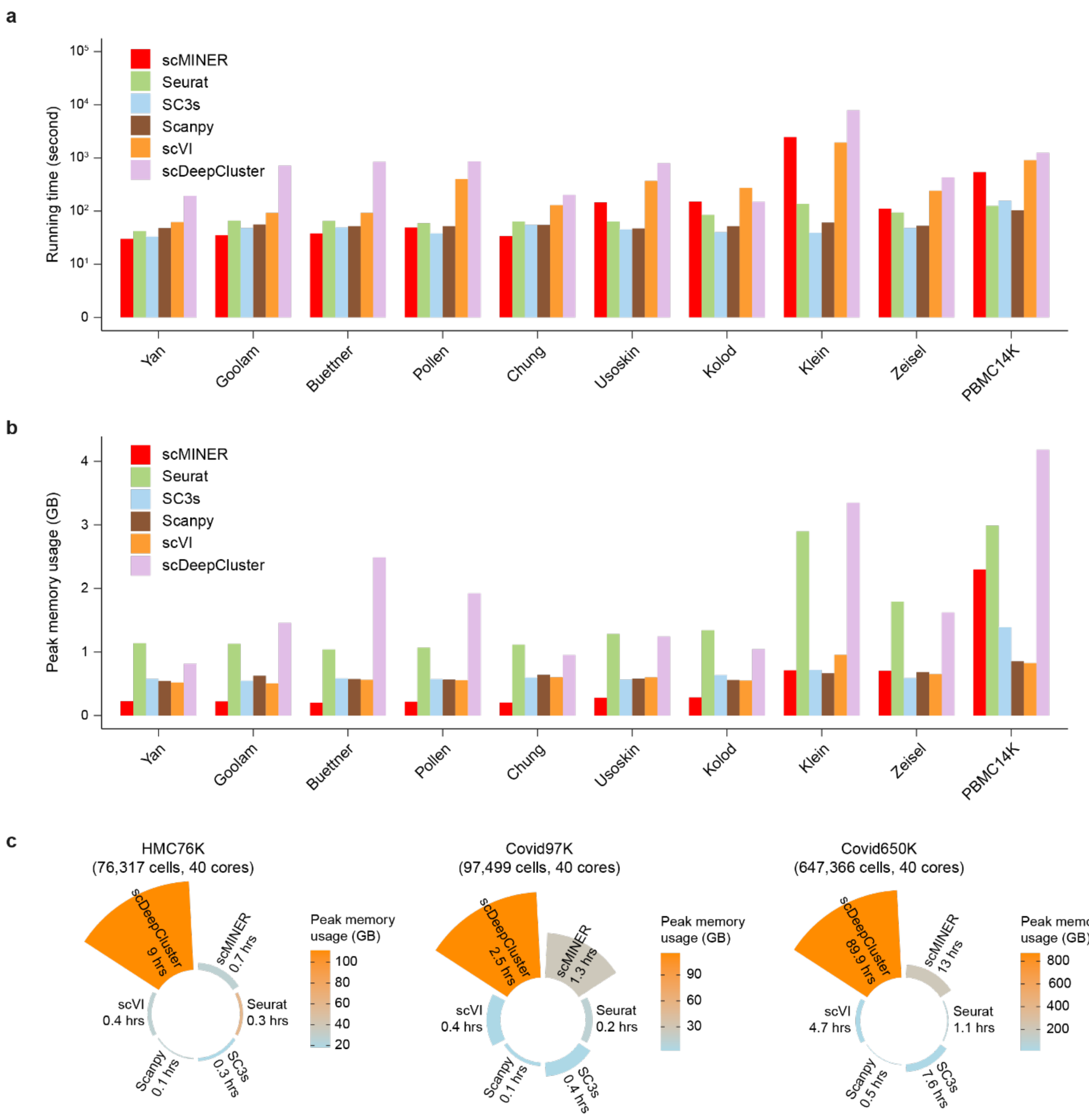

Supplementary Figure 4 | Benchmarking of scMINER for cell clustering efficiency. a,**b**, Comparisons of running time (**a**) and peak memory usage (**b**) by scMINER and other five benchmarked algorithms for the analysis of 10 ground-truth datasets. **c**, Running time and peak memory usage of scMINER against other five methods across three large datasets. All the benchmarks were performed with 40 cores. Source data are provided as a Source Data file.

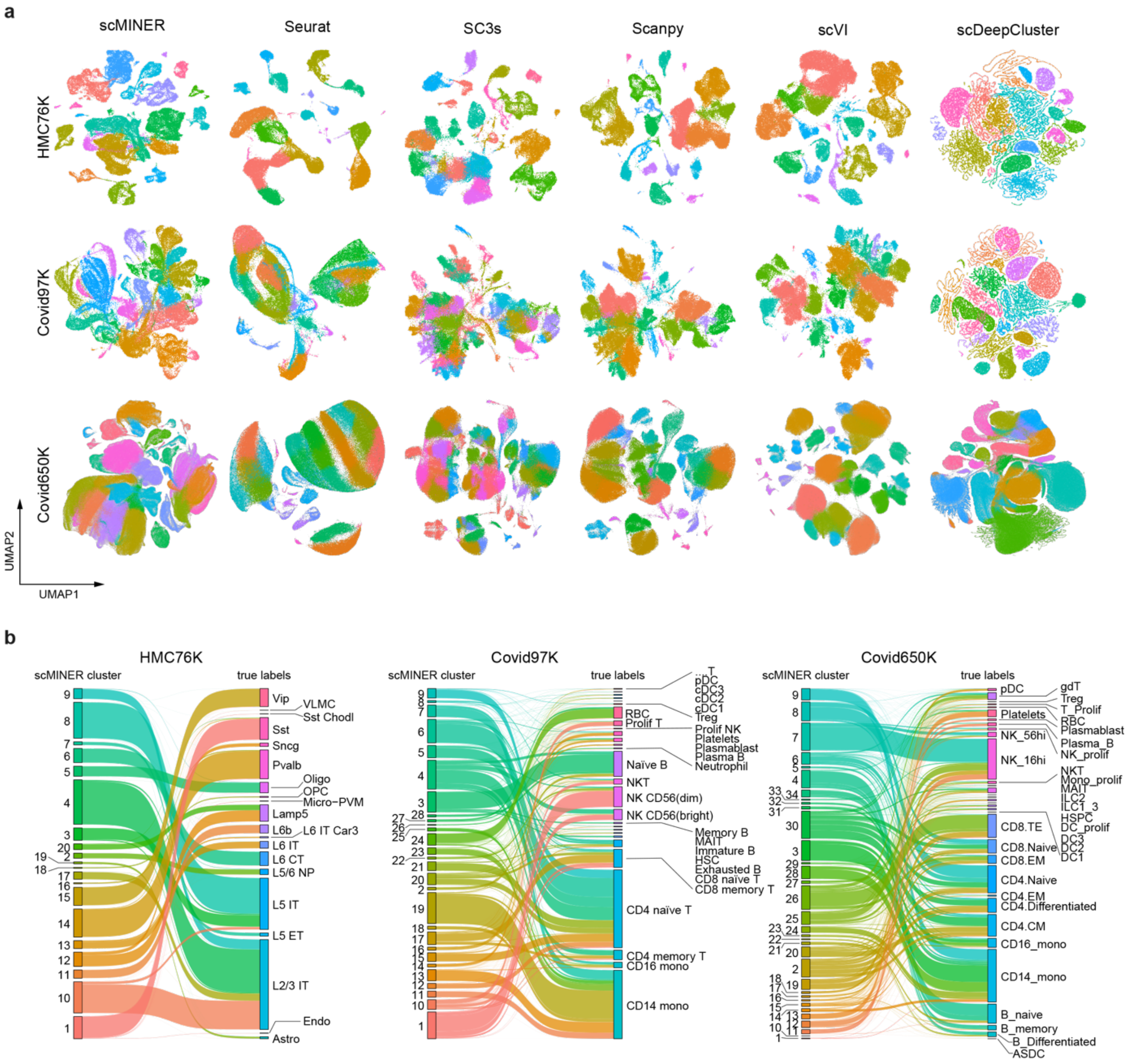

Supplementary Figure 5 | The clustering results of three large datasets. a, UMAPs of three large datasets generated by scMINER and other five benchmarked algorithms. **b**. Sankey diagrams comparing the clusters assigned by scMINER (left) with those reported by data sources. The width of the edges corresponds to the number of cells common to the linked nodes. Source data are provided as a Source Data file.

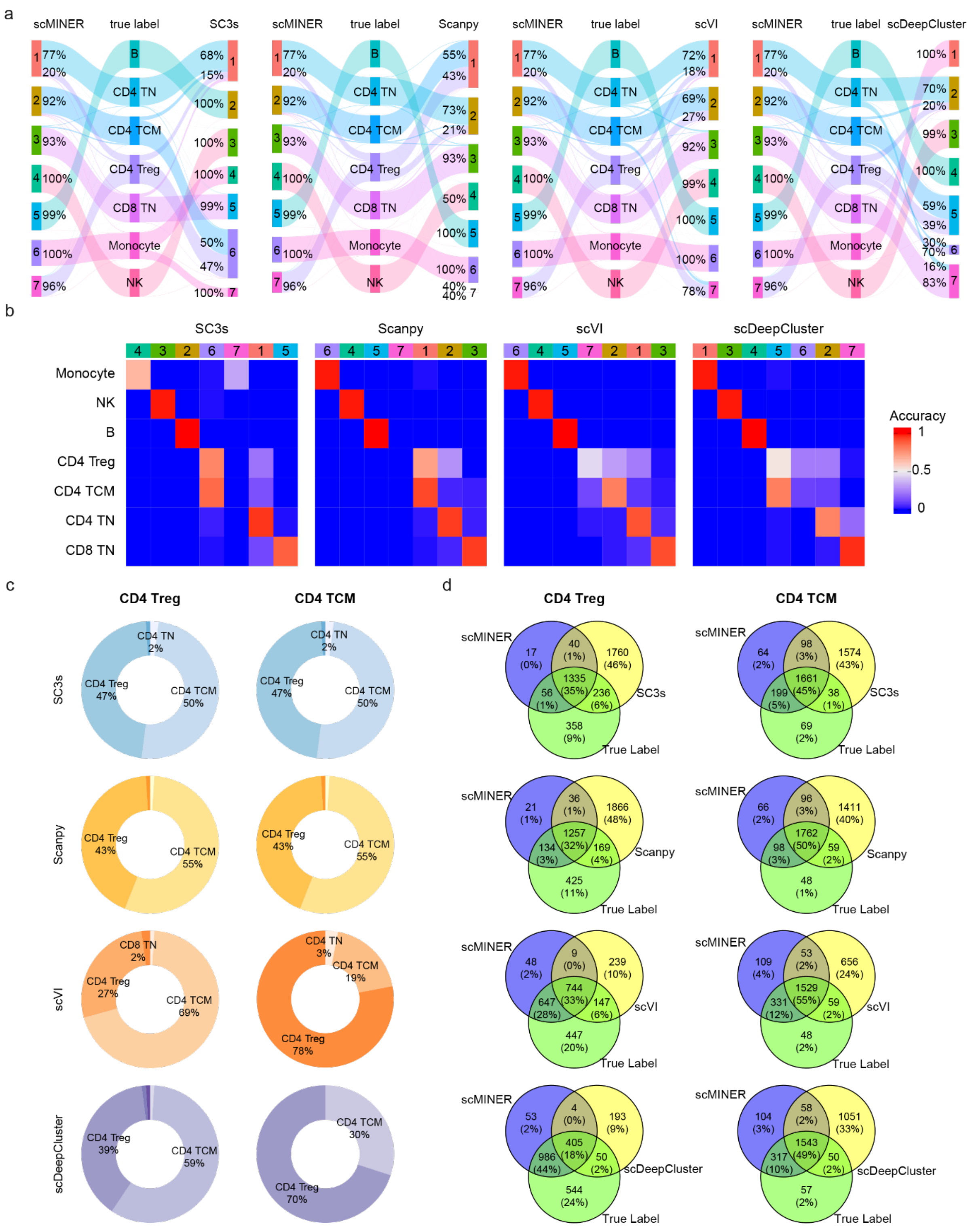

Supplementary Figure 6 | Benchmarking of scMINER against SC3s, Scanpy, scVI and scDeepCluster for distinguishing ambiguous subpopulations in PBMCs. a, Sankey plots showing the overlap between ground-truth labels (middle) and the labels predicted by scMINER

(left) and other four algorithms (right). **b**, Heatmaps comparing the accuracy of clusters predicted by SC3s, Scanpy, scVI and scDeepCluster. **c**, Donut plots indicating the cell composition of major clusters predicted as CD4 Treg (left) and CD5 TCM cells (right) by four algorithms. **d**, Venn diagrams summarizing the overlap between ground-truth labels and predicted labels by scMINER and other four algorithms for CD4 Treg (left) and CD4 TCM cells (right). CD4 Treg: CD4+ regulatory T cells; CD4 TCM: CD4+ central memory T cells; CD4 TN: CD4+ naïve T cells; CD8 TN: CD8+ naïve T cells; NK: natural killer cells. Source data are provided as a Source Data file.

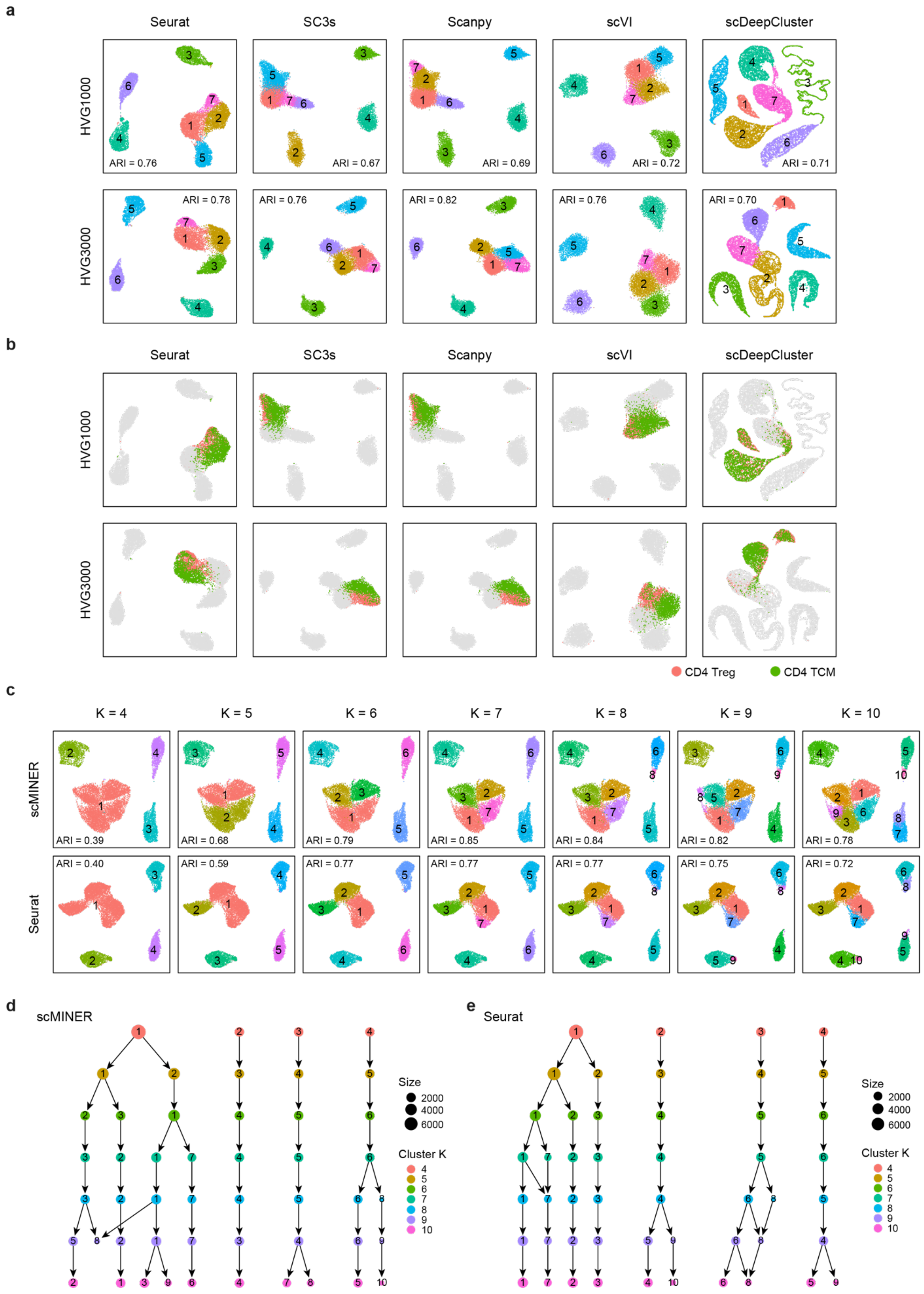
a
Seurat
SC3s
Scanpy
scVI
scDeepCluster
HVG1000
HVG3000
ARI = 0.76
ARI = 0.67
ARI = 0.69
ARI = 0.72
ARI = 0.71
ARI = 0.78
ARI = 0.76
ARI = 0.82
ARI = 0.76
ARI = 0.70
b
Seurat
SC3s
Scanpy
scVI
scDeepCluster
HVG1000
HVG3000
CD4 Treg
CD4 TCM
c
K = 4
K = 5
K = 6
K = 7
K = 8
K = 9
K = 10
scMINER
Seurat
ARI = 0.39
ARI = 0.68
ARI = 0.79
ARI = 0.85
ARI = 0.84
ARI = 0.82
ARI = 0.78
ARI = 0.40
ARI = 0.59
ARI = 0.77
ARI = 0.77
ARI = 0.77
ARI = 0.75
ARI = 0.72
d
scMINER
e
Seurat
Size
2000
4000
6000
Cluster K
4
5
6
7
8
9
10

Supplementary Figure 7 | Impact evaluation of number of highly variable genes and number of clusters on cell clustering accuracy in PBMC14K dataset. a,b, UMAPs of the cell clusters predicted by each algorithm using 1,000 HVGs (top) and 3,000 HVGs (bottom). The projections of CD4 Treg and CD4 TCM cells were highlighted based on ground-truth labels. HVGs: highly variable genes. **c**, UMAPs of the cell clustered predicted by scMINER (top) and Seurat (bottom) with different number of clusters ranging from 4 to 10. **d,e**, Clustering tree plots of scMINER (d) and Seurat (e) showing the flow of cells among clusters as the clustering resolution increases. The size of the nodes denotes the number of cells in each cluster, and the color of the nodes indicates the clustering resolution. Source data are provided as a Source Data file.

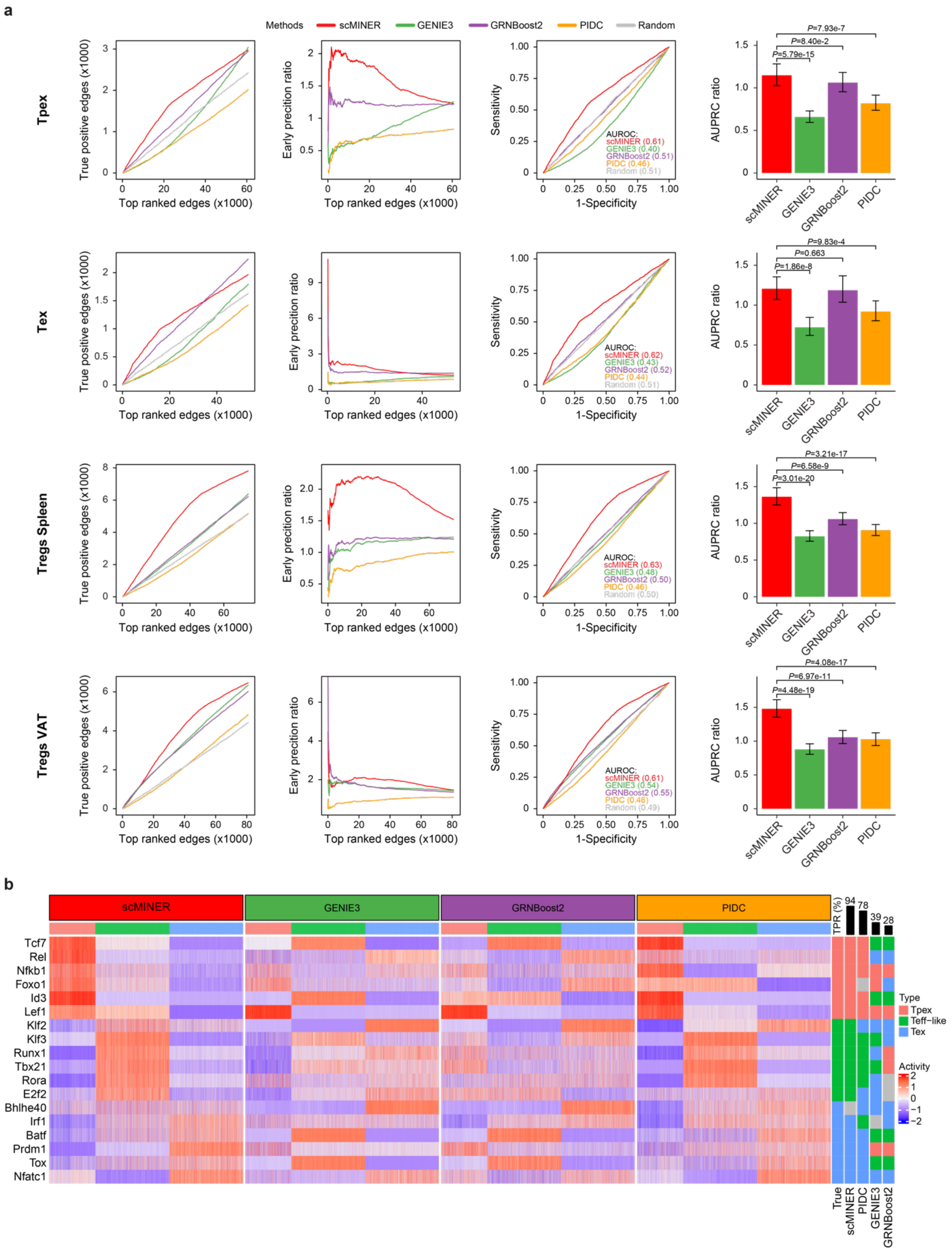
a
Methods scMINER GENIE3 GRNBoost2 PIDC Random
Tpex
Tex
Tregs Spleen
Tregs VAT
True positive edges (x1000)
Top ranked edges (x1000)
Early precition ratio
Sensitivity
1-Specificity
AUROC:
scMINER (0.61)
GENIE3 (0.40)
GRNBoost2 (0.51)
PIDC (0.46)
Random (0.51)
scMINER (0.62)
GENIE3 (0.43)
GRNBoost2 (0.52)
PIDC (0.44)
Random (0.51)
scMINER (0.63)
GENIE3 (0.48)
GRNBoost2 (0.50)
PIDC (0.46)
Random (0.50)
scMINER (0.61)
GENIE3 (0.54)
GRNBoost2 (0.55)
PIDC (0.46)
Random (0.49)
AUPRC ratio
P=7.93e-7
P=8.40e-2
P=5.79e-15
P=9.83e-4
P=0.663
P=1.86e-8
P=3.21e-17
P=6.58e-9
P=3.01e-20
P=4.08e-17
P=6.97e-11
P=4.48e-19
scMINER GENIE3 GRNBoost2 PIDC
b
scMINER GENIE3 GRNBoost2 PIDC
Tcf7
Rel
Nfkb1
Foxo1
Id3
Lef1
Klf2
Klf3
Runx1
Tbx21
Rora
E2f2
Bhlhe40
Irf1
Batf
Prdm1
Tox
Nfatc1
TPR (%) 94 78 39 28
True scMINER PIDC GENIE3 GRNBoost2
Type Tpex Teff-like Tex
Activity 2 1 0 −1 −2

Supplementary Figure 8 | Comparison of scMINER and other three benchmarked algorithms in retrieving the ground-truth TF targets defined by ATAC-seq data and known markers of T cell exhaustion. a, Comparison shows number of true positive predictions as a function of top-ranked edges (first column), early precision ratio (second column), ROC curves (third column), AUPRC ratio for individual TFs (fourth column). Evaluation was performed for progenitor exhausted T cells (Tpex), terminally exhausted T cells (Tex), tissue-specific regulatory T cells (Tregs) from spleen and visceral adipose tissue (VAT). **b,** Evaluation of activity of known markers of T-cells exhaustion based on networks inferred by each evaluated method. The colormap represents activity value, and the annotation on the right corresponds to true-positive rate of predicted markers by each method. Markers were assigned to cell type based on t-test (see methods). Markers without unique assignment to cell type are represented with grey in the annotation. Source data are provided as a Source Data file.

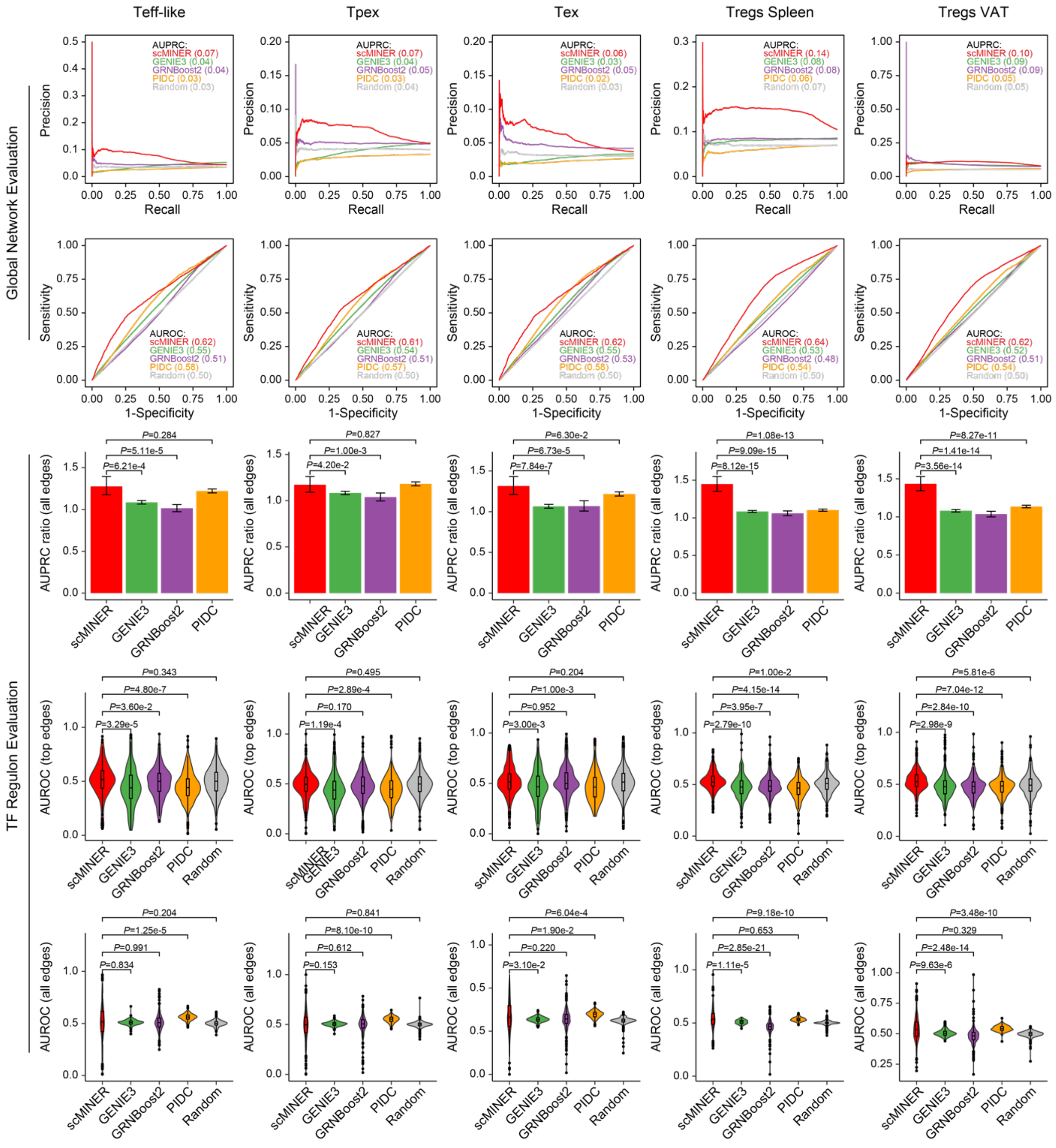

Supplementary Figure 9 | Evaluation of scMINER and three other methods for TF network inference using additional metrics in five ATAC-seq datasets. In global TF network evaluation, scMINER showed increased performance based on precision-recall (first row) and receiver operating characteristics for all edges reported by each method (second row). In the evaluation of individual TF regulons, the performance of scMINER was better or roughly the same compared to alternative methods. Metrics used for TF regulons evaluation: AUPRC ratio for all reported targets,

AUROC values for top predicted and all targets reported by each method. Source data are provided as a Source Data file.

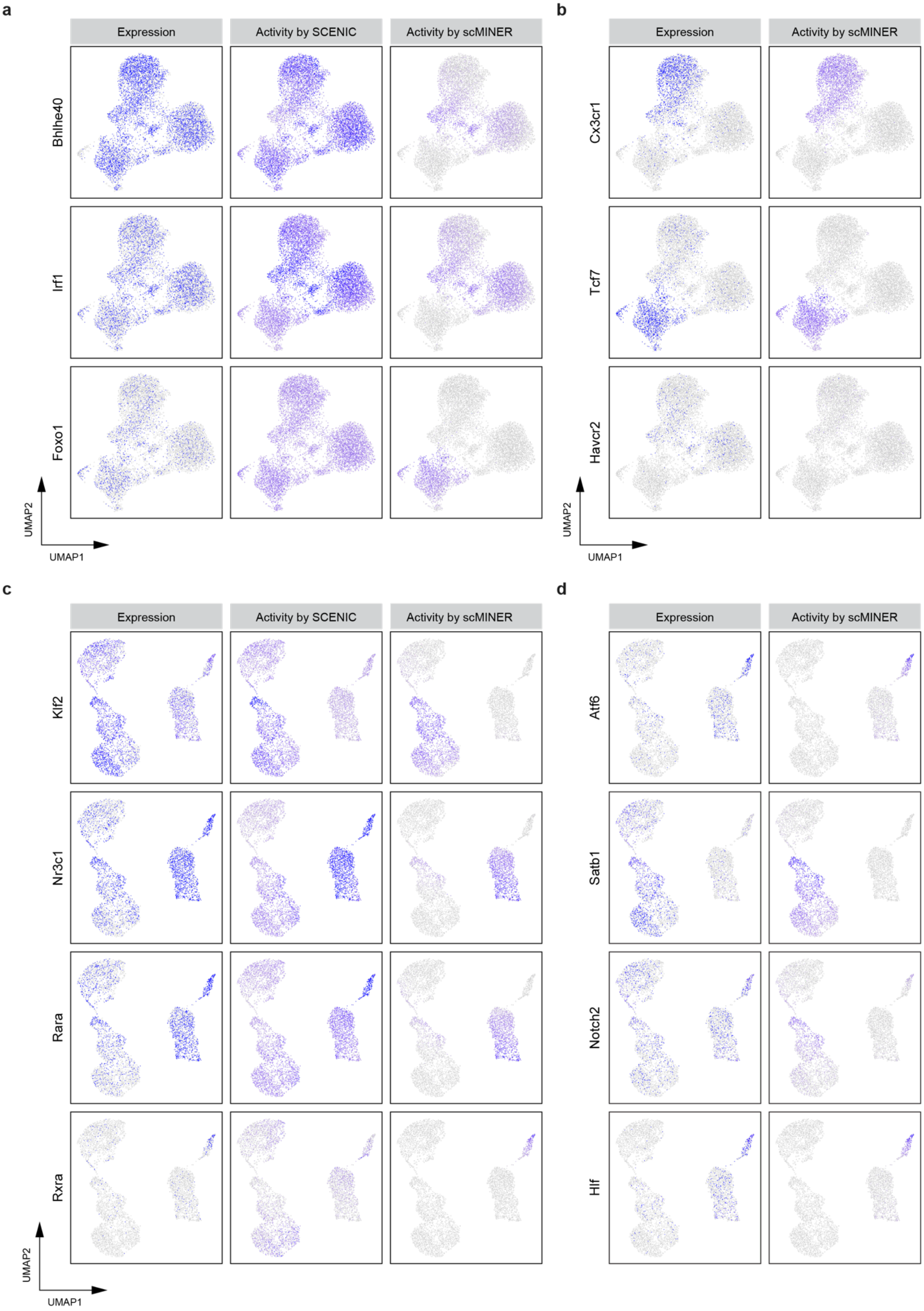
a
Expression
Activity by SCENIC
Activity by scMINER
Bhlhe40
Irf1
Foxo1
UMAP2
UMAP1
b
Expression
Activity by scMINER
Cx3cr1
Tcf7
Havcr2
UMAP2
UMAP1
c
Expression
Activity by SCENIC
Activity by scMINER
Klf2
Nr3c1
Rara
Rxra
UMAP2
UMAP1
d
Expression
Activity by scMINER
Atf6
Satb1
Notch2
Hlf

Supplementary Figure 10 | Comparison of expression and activity of selected TF markers in exhausted T cell (a,b) and tissue-specific Treg (c,d) datasets. Feature plots showing expression and activity computed with SCENIC (when available) and scMINER of selected TF marker genes in exhausted T cells dataset (**a** – Bhlhr40, Irf1, Foxo1, **b** – Cx3cr1, Tcf7, Havcr2) and tissue-specific regulatory T cells dataset (**c** – Klf2, Nr3c1, Rara, Rxra, **d** – Atf6, Satb1, Notch2, Hlf). Activity computed with scMINER helps to compensate for sparsity of single-cell expression values and exhibits more specific signal than activity computed with SCENIC. Source data are provided as a Source Data file.

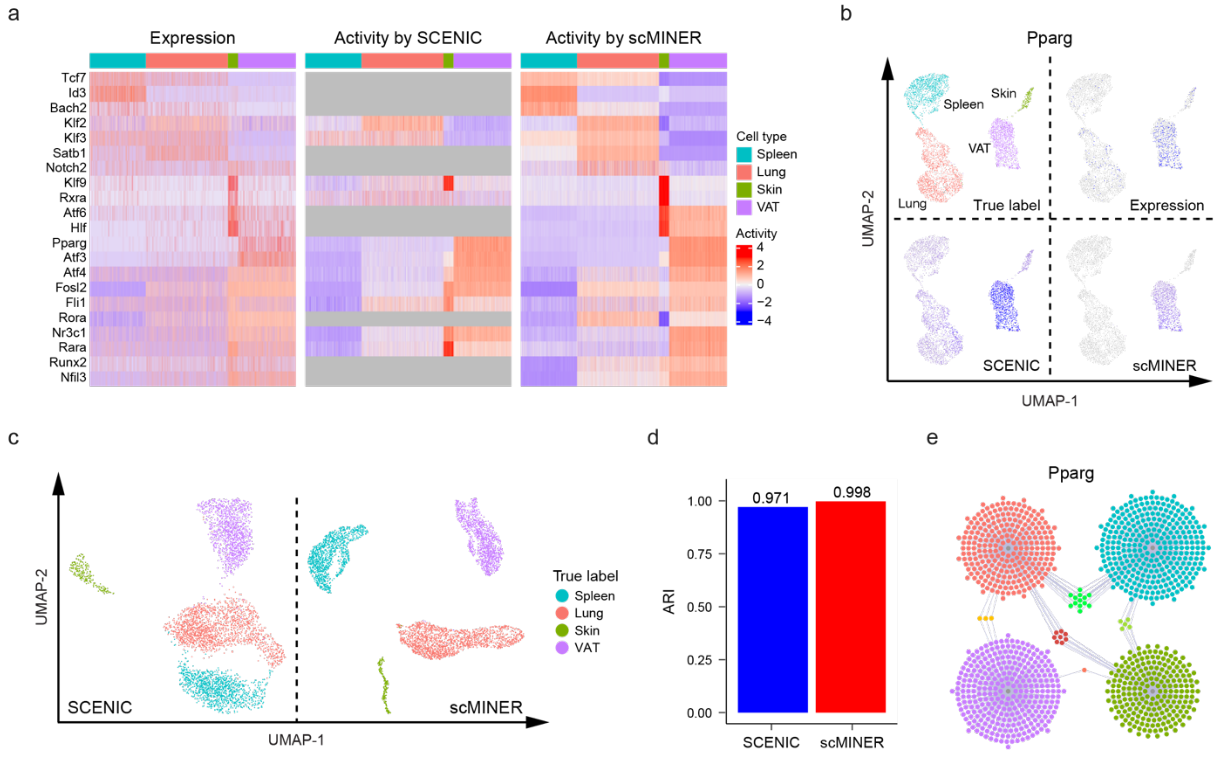

Supplementary Figure 11 | Benchmarking of scMINER against SCENIC for driver identification and activity-based clustering in the tissue-specific Treg dataset. a, Heatmaps showing the expression (left), activity by SCENIC (middle) and scMINER (right) of known markers of tissue-specific Treg cells. **b**, UMAPs of tissue-specific Treg cells highlighting the true labels of cell type (top-left), Pparg expression (top-right), Pparg activity by SCENIC (bottom-left) and scMINER (bottom-right). **c**, UMAPs of activity-based clustering by SCENIC and scMINER. **d**, ARI values of activity-based clustering using SCENIC and scMINER activities. **e**, The regulon rewiring diagram of Pparg among Spleen- (blue), Lung- (red), Skin- (green) and VAT- (purple)specific Treg cells. Source data are provided as a Source Data file.

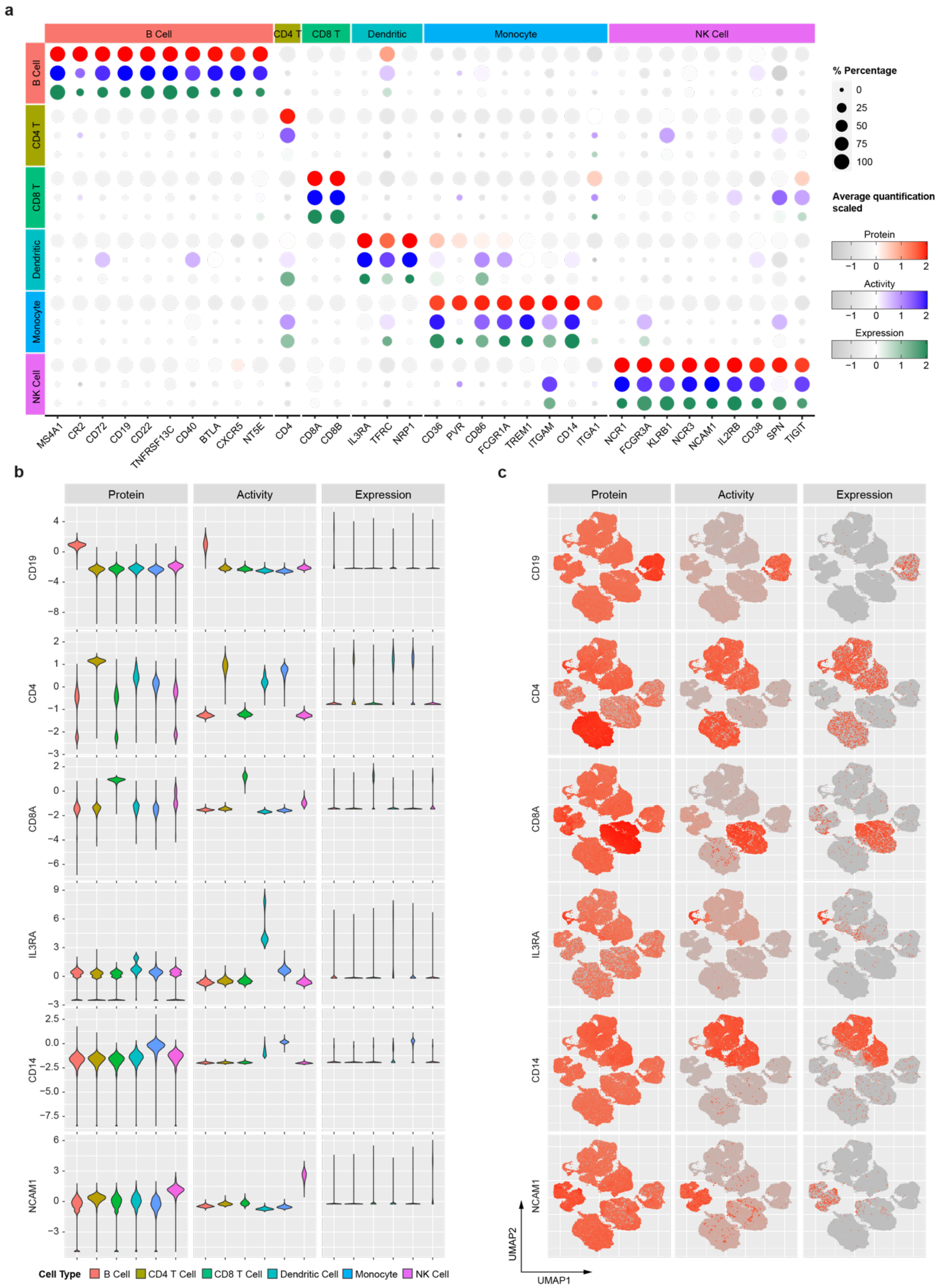
a
B Cell
CD4 T
CD8 T
Dendritic
Monocyte
NK Cell
MS4A1 CR2 CD72 CD19 CD22 TNFRSF13C CD40 BTLA CXCR5 NT5E CD4 CD8A CD8B IL3RA TFRC NRP1 CD36 PVR CD86 FCGR1A TREM1 ITGAM CD14 ITGA1 NCR1 FCGR3A KLRB1 NCR3 NCAM1 IL2RB CD38 SPN TIGIT
% Percentage
0
25
50
75
100
Average quantification scaled
Protein
Activity
Expression
−1 0 1 2
b
Protein
Activity
Expression
CD19
CD4
CD8A
IL3RA
CD14
NCAM1
Cell Type
B Cell
CD4 T Cell
CD8 T Cell
Dendritic Cell
Monocyte
NK Cell
c
Protein
Activity
Expression
CD19
CD4
CD8A
IL3RA
CD14
NCAM1
UMAP2
UMAP1

Supplementary Figure 12 | Benchmarking of scMINER for signaling network inference using an alternative CITE-Seq dataset. **a**, Bubble plot showing the expression, activity and protein quantification by CITE-seq of cell type-specific markers in PBMCs. The cell type-specific markers were defined by differential analysis of protein quantification data, with cutoffs of $P <=$ 0.01 and fold change >= 2. The top 10 markers sorted by fold change were shown if over 10 markers were defined. The size of bubbles is proportional to the percentage of cells with quantification measurements, and the color of bubble is proportional to the scaled quantification measurements. **b**,**c**, Violin plots (**b**) and scatter plots (**c**) showing the distribution of expression, activity by scMINER and protein quantification by CITE-seq of some known cell type-specific markers. Source data are provided as a Source Data file.

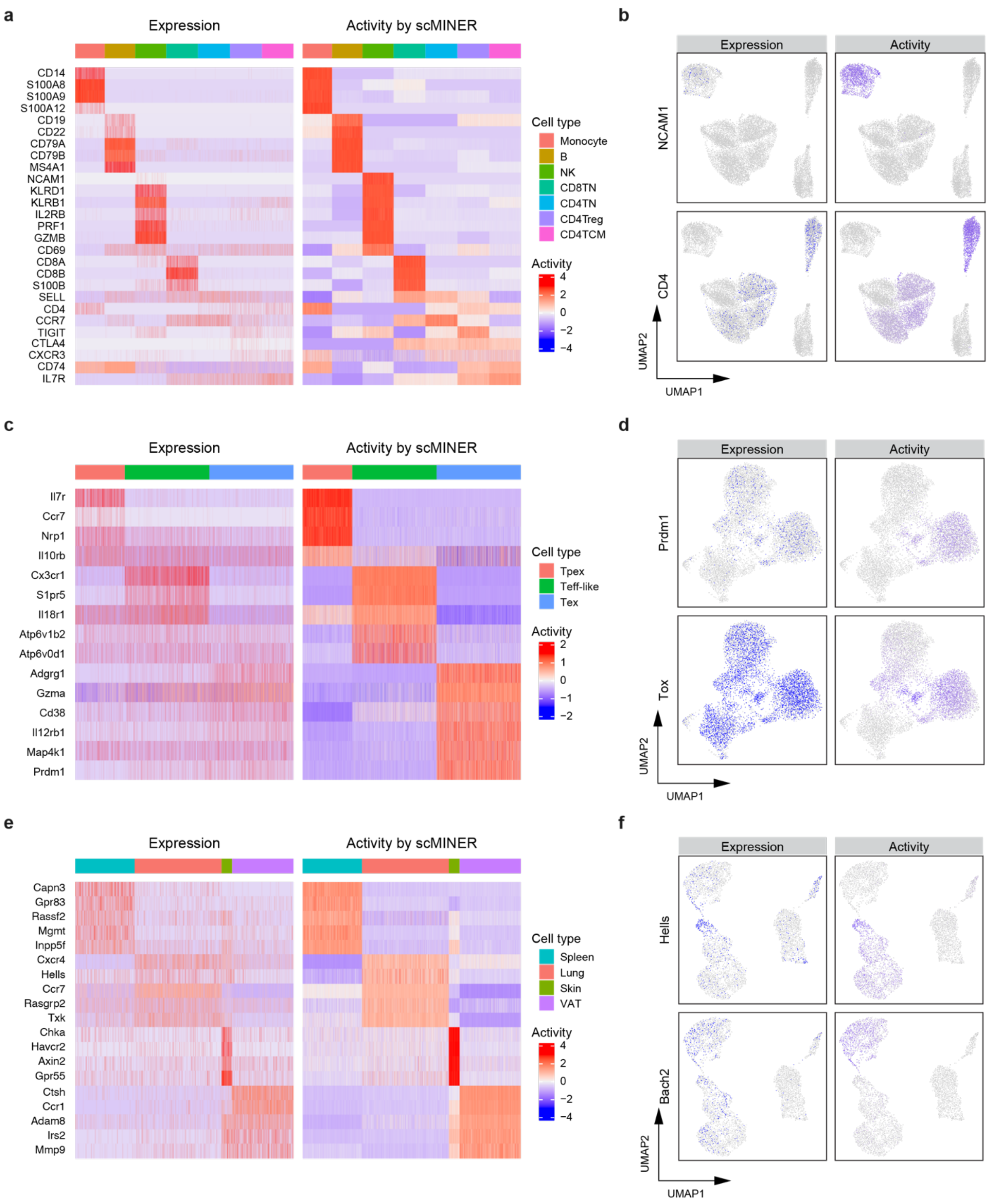

Supplementary Figure 13 | Comparison of the identification of signaling markers from expression data and scMINER-derived activity data in PBMC14K (a,b), exhausted T cells (c,d) and tissue-specific Tregs (e,f) datasets. a,c,d, Heatmaps showing expression (left) and

activity (right) of analyzed markers. **b**,**d**,**f**, Feature plots showing the expression (left) and activity (right) of selected markers in each dataset. Source data are provided as a Source Data file.

Supplementary Tables

Supplementary Table 1 | Summary of datasets used for cell clustering benchmark.

Dataset	Protocol	Size	Classes	Species	Tissue	Accession ID
Yan[1]	Tang	124	8	Human	Embryonic stem	GSE36552
Goolam[2]	Smart-Seq2	124	5	Mouse	Development embryos	E-MTAB-3321
Buettner[3]	C1	182	3	Mouse	Embryonic stem	E-MTAB-2805
Pollen[4]	SMARTer	249	11	Human	Cerebral cortex	SRP041736
Chung[5]	SMARTer	317	5	Human	Breast cancer	GSE75688
Usoskin[6]	STRT-seq	622	4	Mouse	Sensory neurons	GSE59739
Kolod[7]	SMARTer	704	3	Mouse	Embryonic stem	E-MTAB-2600
Klein[8]	inDrop	2,717	4	Mouse	Embryonic Stem	GSE65525
Zeisel[9]	STRT-seq	2,903	7	Mouse	Cortex, hippocampus	GSE60361
PBMC14K[10]	10x Genomics	13,605	7	Human	Peripheral blood	SRP073767
HMC76K[11]	10x Genomics	76,533	20	Human	Motor cortex	Azimuth
Covid97K[12]	10x Genomics	97,499	28	Human	Peripheral blood	E-MTAB-10026
Covid650K[13]	10x Genomics	647,366	51	Human	Peripheral blood	EGAD00001007982

Supplementary Table 2 | Summary of datasets used for network inference and activity estimation benchmarks.

Accession ID	Data type	Cell types	Protocol
GSE122712	scRNA-seq	$CD8^+$ T cells from chronic infection[14]	10x Genomics
GSE130879	scRNA-seq	Tissue (spleen, lung, skin, and VAT) Treg cells[15]	10x Genomics
GSE123236	ATAC-seq	Tpex and Tex in LCMV infection[14]	Bulk
GSE112731	ATAC-seq	Tissue-specific (spleen and VAT) Treg cells[16]	Bulk
GSE221321	Perturb-seq	THP-1 cells[17]	10x Genomics
GSE218988	CROP-seq	$CD8^+$ T cells from CRISPRi/a screens[18]	10x Genomics
GSE213282	CITE-seq	PBMCs (B, CD4T, CD8T, Monocyte, NK)[19]	10x Genomics (5’)
GSE164378	CITE-seq	PBMCs (B, CD4T, CD8T, DC, Monocyte, NK)[20]	10x Genomics (3’)

Supplementary Table 3 | The known markers of PBMCs, CD8+ exhausted T cells and tissue-specific Tregs.

Dataset	Cell Type	Marker Type	Marker List
PBMC14K	Monocyte	TF	CEBPA[21], CEBPB[21], SPI1[22]
		SIG	CD14[23], S100A8[23], S100A9[23], S100A12[24]
	B	TF	IRF8[25]
		SIG	CD19[26], CD22[27], CD79A[28], CD79B[28], MS4A1[29]
	NK	TF	TBX21[30], PRDM1[31]
		SIG	NCAM1[32], KLRD1[33], KLRB1[34], IL2RB[35], PRF1[34], GZMB[36], CD69[37], NFATC3[38,39]
	CD8TN	TF	EOMES[40,41], LEF1[42], TCF7[43]
		SIG	CD8A, CD8B, S100B[44], NFATC3[38,39]
	CD4TN	TF	SATB1[45]
		SIG	CD4, SELL[46], CCR7[47]
	CD4Treg	TF	FOXP3[48], GATA3[49]
		SIG	CD4, TIGIT[50], CTLA4[51], CXCR3[52]
	CD4TCM	TF	KLF2[53-55], NFATC3[38,39]
		SIG	CD74[56], IL7R[57]
Exhausted T cells	Tpex	TF	Tcf7[58], Id3[58], Rel[59], Foxo1[59], Nfkb1[59], Lef1[60]
		SIG	Il7r[61], Ccr7[62], Nrp1[63], Il10rb[64]
	Teff-like	TF	Klf2[65], Klf3[65], Runx1[66], Tbx21[30], Rora[67]
		SIG	Cx3cr1[68], S1pr5[69], Il18r1[70], Atp6v1b2[71], Atp6v0d1[71]
	Tex	TF	E2f2[72], Bhlhe40[73], Irf1[74], Batf[75], Tox[76], Nfatc1[38]
		SIG	Adgrg1[77], Gzma[78], Cd38[79], Il12rb1[38], Map4k1[80], Prdm1[81]
Tissue-specific Tregs	Spleen	TF	Tcf7[82], Id3[83], Bach2[84]
		SIG	Capn3[85,86], Gpr83[86,87], Rassf2[88], Mgmt[89], Inpp5f[90]
	Lung	TF	Klf2[91], Klf3[91], Satb1[92], Notch2[93]
		SIG	Cxcr4[94], Hells[95], Ccr7[96], Rasgrp2[97], Txk[98]
	Skin	TF	Klf9[99], Rxra[99], Atf6[99], Hlf[99]
		SIG	Chka[100], Havcr2[99], Axin2[99], Gpr55[99]
	VAT	TF	Pparg[99], Atf3[99], Atf4[99], Fosl2[99], Fli1[16], Rora[99], Nr3c1[99], Rara[16], Runx2[99], Nfil3[99],
		SIG	Ctsh[99], Ccr1[99], Adam8[99], Irs2[99], Mmp9[99]

Supplementary References

1. Yan, L. *et al.* Single-cell RNA-Seq profiling of human preimplantation embryos and embryonic stem cells. *Nat Struct Mol Biol* **20**, 1131-9 (2013).
2. Goolam, M. *et al.* Heterogeneity in Oct4 and Sox2 Targets Biases Cell Fate in 4-Cell Mouse Embryos. *Cell* **165**, 61-74 (2016).
3. Buettner, F. *et al.* Computational analysis of cell-to-cell heterogeneity in single-cell RNA-sequencing data reveals hidden subpopulations of cells. *Nature Biotechnology* **33**, 155-160 (2015).
4. Pollen, A.A. *et al.* Low-coverage single-cell mRNA sequencing reveals cellular heterogeneity and activated signaling pathways in developing cerebral cortex. *Nature Biotechnology* **32**, 1053-1058 (2014).
5. Chung, W. *et al.* Single-cell RNA-seq enables comprehensive tumour and immune cell profiling in primary breast cancer. *Nature Communications* **8**, 15081 (2017).
6. Usoskin, D. *et al.* Unbiased classification of sensory neuron types by large-scale single-cell RNA sequencing. *Nature Neuroscience* **18**, 145-153 (2015).
7. Kolodziejczyk, Aleksandra A. *et al.* Single Cell RNA-Sequencing of Pluripotent States Unlocks Modular Transcriptional Variation. *Cell Stem Cell* **17**, 471-485 (2015).
8. Klein, Allon M. *et al.* Droplet Barcoding for Single-Cell Transcriptomics Applied to Embryonic Stem Cells. *Cell* **161**, 1187-1201 (2015).
9. Zeisel, A. *et al.* Cell types in the mouse cortex and hippocampus revealed by single-cell RNA-seq. *Science* **347**, 1138-1142 (2015).
10. Zheng, G.X.Y. *et al.* Massively parallel digital transcriptional profiling of single cells. *Nature Communications* **8**, 14049 (2017).
11. Bakken, T.E. *et al.* Comparative cellular analysis of motor cortex in human, marmoset and mouse. *Nature* **598**, 111-119 (2021).
12. Stephenson, E. *et al.* Single-cell multi-omics analysis of the immune response in COVID-19. *Nature Medicine* **27**, 904-916 (2021).
13. Godoy-Tena, G. *et al.* Epigenetic and transcriptomic reprogramming in monocytes of severe COVID-19 patients reflects alterations in myeloid differentiation and the influence of inflammatory cytokines. *Genome Medicine* **14**, 134 (2022).
14. Miller, B.C. *et al.* Subsets of exhausted $CD8^{+}$ T cells differentially mediate tumor control and respond to checkpoint blockade. *Nat Immunol* **20**, 326-336 (2019).
15. Delacher, M. *et al.* Precursors for Nonlymphoid-Tissue Treg Cells Reside in Secondary Lymphoid Organs and Are Programmed by the Transcription Factor BATF. *Immunity* **52**, 295-312 e11 (2020).
16. DiSpirito, J.R. *et al.* Molecular diversification of regulatory T cells in nonlymphoid tissues. *Sci Immunol* **3**(2018).
17. Yan, Y., Dai, W. & Mei, Q. Multicentric Glioma: An Ideal Model to Reveal the Mechanism of Glioma. *Front Oncol* **12**, 798018 (2022).

18. McCutcheon, S.R. *et al.* Transcriptional and epigenetic regulators of human CD8(+) T cell function identified through orthogonal CRISPR screens. *Nat Genet* **55**, 2211-2223 (2023).
19. Nettersheim, F.S. *et al.* Titration of 124 antibodies using CITE-Seq on human PBMCs. *Sci Rep* **12**, 20817 (2022).
20. Hao, Y. *et al.* Integrated analysis of multimodal single-cell data. *Cell* **184**, 3573-3587 e29 (2021).
21. Kucinski, I. *et al.* Interactions between lineage-associated transcription factors govern haematopoietic progenitor states. *The EMBO journal* **39**, e104983 (2020).
22. Scott, E.W., Simon, M.C., Anastasi, J. & Singh, H. Requirement of transcription factor PU.1 in the development of multiple hematopoietic lineages. *Science* **265**, 1573-7 (1994).
23. Kapellos, T.S. *et al.* Human Monocyte Subsets and Phenotypes in Major Chronic Inflammatory Diseases. *Front Immunol* **10**, 2035 (2019).
24. Zhao, F. *et al.* S100A9 a new marker for monocytic human myeloid-derived suppressor cells. *Immunology* **136**, 176-83 (2012).
25. Wang, H. *et al.* IRF8 regulates B-cell lineage specification, commitment, and differentiation. *Blood* **112**, 4028-38 (2008).
26. Wang, K., Wei, G. & Liu, D. CD19: a biomarker for B cell development, lymphoma diagnosis and therapy. *Exp Hematol Oncol* **1**, 36 (2012).
27. Clark, E.A. & Giltiay, N.V. CD22: A Regulator of Innate and Adaptive B Cell Responses and Autoimmunity. *Front Immunol* **9**, 2235 (2018).
28. Mason, D.Y. *et al.* CD79a: a novel marker for B-cell neoplasms in routinely processed tissue samples. *Blood* **86**, 1453-9 (1995).
29. Pavlasova, G. & Mraz, M. The regulation and function of CD20: an "enigma" of B-cell biology and targeted therapy. *Haematologica* **105**, 1494-1506 (2020).
30. Intlekofer, A.M. *et al.* Effector and memory CD8+ T cell fate coupled by T-bet and eomesodermin. *Nat Immunol* **6**, 1236-44 (2005).
31. Kallies, A. *et al.* A role for Blimp1 in the transcriptional network controlling natural killer cell maturation. *Blood* **117**, 1869-79 (2011).
32. Poli, A. *et al.* CD56bright natural killer (NK) cells: an important NK cell subset. *Immunology* **126**, 458-65 (2009).
33. Gunturi, A., Berg, R.E. & Forman, J. The role of CD94/NKG2 in innate and adaptive immunity. *Immunol Res* **30**, 29-34 (2004).
34. Fan, C. *et al.* PRF1 is a prognostic marker and correlated with immune infiltration in head and neck squamous cell carcinoma. *Transl Oncol* **14**, 101042 (2021).
35. Fernandez, I.Z. *et al.* A novel human IL2RB mutation results in T and NK cell-driven immune dysregulation. *J Exp Med* **216**, 1255-1267 (2019).
36. Crinier, A. *et al.* Single-cell profiling reveals the trajectories of natural killer cell differentiation in bone marrow and a stress signature induced by acute myeloid leukemia. *Cell Mol Immunol* **18**, 1290-1304 (2021).

37. Borrego, F., Robertson, M.J., Ritz, J., Pena, J. & Solana, R. CD69 is a stimulatory receptor for natural killer cell and its cytotoxic effect is blocked by CD94 inhibitory receptor. *Immunology* **97**, 159-65 (1999).
38. Klein-Hessling, S. *et al.* NFATc1 controls the cytotoxicity of CD8(+) T cells. *Nat Commun* **8**, 511 (2017).
39. Rengarajan, J., Tang, B. & Glimcher, L.H. NFATc2 and NFATc3 regulate T(H)2 differentiation and modulate TCR-responsiveness of naive T(H)cells. *Nat Immunol* **3**, 48-54 (2002).
40. Zhang, P. *et al.* Eomesodermin promotes the development of type 1 regulatory T (T(R)1) cells. *Sci Immunol* **2**(2017).
41. Llao-Cid, L. *et al.* EOMES is essential for antitumor activity of CD8(+) T cells in chronic lymphocytic leukemia. *Leukemia* **35**, 3152-3162 (2021).
42. van der Leun, A.M., Thommen, D.S. & Schumacher, T.N. CD8(+) T cell states in human cancer: insights from single-cell analysis. *Nat Rev Cancer* **20**, 218-232 (2020).
43. Escobar, G., Mangani, D. & Anderson, A.C. T cell factor 1: A master regulator of the T cell response in disease. *Sci Immunol* **5**(2020).
44. Steiner, J. *et al.* Human CD8(+) T cells and NK cells express and secrete S100B upon stimulation. *Brain Behav Immun* **25**, 1233-41 (2011).
45. Kakugawa, K. *et al.* Essential Roles of SATB1 in Specifying T Lymphocyte Subsets. *Cell Rep* **19**, 1176-1188 (2017).
46. Cano-Gamez, E. *et al.* Single-cell transcriptomics identifies an effectorness gradient shaping the response of CD4(+) T cells to cytokines. *Nat Commun* **11**, 1801 (2020).
47. Bjorkdahl, O. *et al.* Characterization of CC-chemokine receptor 7 expression on murine T cells in lymphoid tissues. *Immunology* **110**, 170-9 (2003).
48. Rudensky, A.Y. Regulatory T cells and Foxp3. *Immunological reviews* **241**, 260-268 (2011).
49. Kiaf, B., Bode, K., Schuster, C. & Kissler, S. Gata3 is detrimental to regulatory T cell function in autoimmune diabetes. *bioRxiv* (2023).
50. Joller, N. *et al.* Treg cells expressing the coinhibitory molecule TIGIT selectively inhibit proinflammatory Th1 and Th17 cell responses. *Immunity* **40**, 569-81 (2014).
51. Walker, L.S. Treg and CTLA-4: two intertwining pathways to immune tolerance. *J Autoimmun* **45**, 49-57 (2013).
52. Moreno Ayala, M.A. *et al.* CXCR3 expression in regulatory T cells drives interactions with type I dendritic cells in tumors to restrict CD8(+) T cell antitumor immunity. *Immunity* **56**, 1613-1630 e5 (2023).
53. Takada, K. *et al.* Kruppel-like factor 2 is required for trafficking but not quiescence in postactivated T cells. *J Immunol* **186**, 775-83 (2011).
54. Weinreich, M.A. *et al.* KLF2 transcription-factor deficiency in T cells results in unrestrained cytokine production and upregulation of bystander chemokine receptors. *Immunity* **31**, 122-30 (2009).
55. Sebzda, E., Zou, Z., Lee, J.S., Wang, T. & Kahn, M.L. Transcription factor KLF2 regulates the migration of naive T cells by restricting chemokine receptor expression patterns. *Nat Immunol* **9**, 292-300 (2008).

56. Zhang, L. *et al.* CD74 is a functional MIF receptor on activated CD4+ T cells. *Cellular and Molecular Life Sciences* **81**, 296 (2024).
57. Colpitts, S.L., Dalton, N.M. & Scott, P. IL-7 receptor expression provides the potential for long-term survival of both CD62Lhigh central memory T cells and Th1 effector cells during Leishmania major infection. *J Immunol* **182**, 5702-11 (2009).
58. Utzschneider, D.T. *et al.* Early precursor T cells establish and propagate T cell exhaustion in chronic infection. *Nat Immunol* **21**, 1256-1266 (2020).
59. Liu, Z. *et al.* Progenitor-like exhausted SPRY1(+)CD8(+) T cells potentiate responsiveness to neoadjuvant PD-1 blockade in esophageal squamous cell carcinoma. *Cancer Cell* **41**, 1852-1870 e9 (2023).
60. Tsui, C. *et al.* MYB orchestrates T cell exhaustion and response to checkpoint inhibition. *Nature* **609**, 354-360 (2022).
61. Andreatta, M. *et al.* Interpretation of T cell states from single-cell transcriptomics data using reference atlases. *Nat Commun* **12**, 2965 (2021).
62. Zehn, D., Thimme, R., Lugli, E., de Almeida, G.P. & Oxenius, A. 'Stem-like' precursors are the fount to sustain persistent CD8(+) T cell responses. *Nat Immunol* **23**, 836-847 (2022).
63. Ni, L. Potential mechanisms of cancer stem-like progenitor T-cell bio-behaviours. *Clin Transl Med* **14**, e1817 (2024).
64. Park, J.H., Kang, I. & Lee, H.K. The immune landscape of high-grade brain tumor after treatment with immune checkpoint blockade. *Front Immunol* **13**, 1044544 (2022).
65. Hart, G.T., Hogquist, K.A. & Jameson, S.C. Kruppel-like factors in lymphocyte biology. *J Immunol* **188**, 521-6 (2012).
66. Korinfskaya, S., Parameswaran, S., Weirauch, M.T. & Barski, A. Runx Transcription Factors in T Cells-What Is Beyond Thymic Development? *Front Immunol* **12**, 701924 (2021).
67. Neyens, D. *et al.* HELIOS-expressing human CD8 T cells exhibit limited effector functions. *Front Immunol* **14**, 1308539 (2023).
68. Yamauchi, T. *et al.* T-cell CX3CR1 expression as a dynamic blood-based biomarker of response to immune checkpoint inhibitors. *Nat Commun* **12**, 1402 (2021).
69. Im, S.J., Konieczny, B.T., Hudson, W.H., Masopust, D. & Ahmed, R. PD-1+ stemlike CD8 T cells are resident in lymphoid tissues during persistent LCMV infection. *Proc Natl Acad Sci U S A* **117**, 4292-4299 (2020).
70. Hudson, W.H. *et al.* Proliferating Transitory T Cells with an Effector-like Transcriptional Signature Emerge from PD-1(+) Stem-like CD8(+) T Cells during Chronic Infection. *Immunity* **51**, 1043-1058 e4 (2019).
71. Fernandez-Garcia, J. *et al.* CD8(+) T cell metabolic rewiring defined by scRNA-seq identifies a critical role of ASNS expression dynamics in T cell differentiation. *Cell Rep* **41**, 111639 (2022).
72. Blake, M.K., O'Connell, P. & Aldhamen, Y.A. Fundamentals to therapeutics: Epigenetic modulation of CD8(+) T Cell exhaustion in the tumor microenvironment. *Front Cell Dev Biol* **10**, 1082195 (2022).

73. Wu, J.E. *et al.* In vitro modeling of CD8(+) T cell exhaustion enables CRISPR screening to reveal a role for BHLHE40. *Sci Immunol* **8**, eade3369 (2023).
74. Beltra, J.C. *et al.* Developmental Relationships of Four Exhausted CD8(+) T Cell Subsets Reveals Underlying Transcriptional and Epigenetic Landscape Control Mechanisms. *Immunity* **52**, 825-841 e8 (2020).
75. Chen, Y. *et al.* BATF regulates progenitor to cytolytic effector CD8(+) T cell transition during chronic viral infection. *Nat Immunol* **22**, 996-1007 (2021).
76. Bordon, Y. TOX for tired T cells. *Nat Rev Immunol* **19**, 476 (2019).
77. Mei, Y. *et al.* Identifying ADGRG1 as a specific marker for tumor-reactive T cells in acute myeloid leukemia. *Exp Hematol Oncol* **13**, 92 (2024).
78. Zhang, C. *et al.* Prioritizing exhausted T cell marker genes highlights immune subtypes in pan-cancer. *iScience* **26**, 106484 (2023).
79. Reolo, M.J.Y. *et al.* CD38 marks the exhausted CD8(+) tissue-resident memory T cells in hepatocellular carcinoma. *Front Immunol* **14**, 1182016 (2023).
80. Si, J. *et al.* Hematopoietic Progenitor Kinase1 (HPK1) Mediates T Cell Dysfunction and Is a Druggable Target for T Cell-Based Immunotherapies. *Cancer Cell* **38**, 551-566 e11 (2020).
81. Welsh, R.M. Blimp hovers over T cell immunity. *Immunity* **31**, 178-80 (2009).
82. Zheng, M. *et al.* Transcription factor TCF-1 regulates the functions, but not the development, of lymphoid tissue inducer subsets in different tissues. *Cell Rep* **42**, 112924 (2023).
83. Frias, A.B., Jr. *et al.* The Transcriptional Regulator Id2 Is Critical for Adipose-Resident Regulatory T Cell Differentiation, Survival, and Function. *J Immunol* **203**, 658-664 (2019).
84. Tsukumo, S. *et al.* Bach2 maintains T cells in a naive state by suppressing effector memory-related genes. *Proc Natl Acad Sci U S A* **110**, 10735-40 (2013).
85. Leber, A., Hontecillas, R., Zoccoli-Rodriguez, V. & Bassaganya-Riera, J. Activation of LANCL2 by BT-11 ameliorates IBD by supporting regulatory T cell stability through immunometabolic mechanisms. *Inflammatory Bowel Diseases* **24**, 1978-1991 (2018).
86. Layland, L.E. *et al.* Pronounced phenotype in activated regulatory T cells during a chronic helminth infection. *J Immunol* **184**, 713-24 (2010).
87. Zemmour, D. *et al.* Single-cell gene expression reveals a landscape of regulatory T cell phenotypes shaped by the TCR. *Nat Immunol* **19**, 291-301 (2018).
88. Abedi, M. *et al.* Aberrant TNF signaling in pancreatic lymph nodes of patients with Type 1 Diabetes. *bioRxiv*, 2024.05. 31.596885 (2024).
89. Amoozgar, Z. *et al.* Targeting Treg cells with GITR activation alleviates resistance to immunotherapy in murine glioblastomas. *Nat Commun* **12**, 2582 (2021).
90. Collazo, M.M., Paraiso, K.H., Park, M.Y., Hazen, A.L. & Kerr, W.G. Lineage extrinsic and intrinsic control of immunoregulatory cell numbers by SHIP. *Eur J Immunol* **42**, 1785-95 (2012).
91. Wittner, J. & Schuh, W. Kruppel-like Factor 2 (KLF2) in Immune Cell Migration. *Vaccines (Basel)* **9**(2021).

92. Zagorulya, M. *et al.* Tissue-specific abundance of interferon-gamma drives regulatory T cells to restrain DC1-mediated priming of cytotoxic T cells against lung cancer. *Immunity* **56**, 386-405 e10 (2023).
93. Fan, H.M. *et al.* Long-term consequences of regulatory T-cell-specific knockout of Notch2 in immune homeostasis. *Int Immunopharmacol* **124**, 111069 (2023).
94. Huppert, L.A. *et al.* Tissue-specific Tregs in cancer metastasis: opportunities for precision immunotherapy. *Cell Mol Immunol* **19**, 33-45 (2022).
95. He, X. *et al.* Identification of immune-associated signatures and potential therapeutic targets for pulmonary arterial hypertension. *J Cell Mol Med* **27**, 3864-3877 (2023).
96. Trujillo, G., Hartigan, A.J. & Hogaboam, C.M. T regulatory cells and attenuated bleomycin-induced fibrosis in lungs of CCR7-/- mice. *Fibrogenesis Tissue Repair* **3**, 18 (2010).
97. Strutt, T.M. *et al.* IL-15 supports the generation of protective lung-resident memory CD4 T cells. *Mucosal Immunol* **11**, 668-680 (2018).
98. Shah, K., Al-Haidari, A., Sun, J. & Kazi, J.U. T cell receptor (TCR) signaling in health and disease. *Signal Transduct Target Ther* **6**, 412 (2021).
99. Munoz-Rojas, A.R. & Mathis, D. Tissue regulatory T cells: regulatory chameleons. *Nat Rev Immunol* **21**, 597-611 (2021).
100. Wu, D. *et al.* A single-cell atlas of IL-23 inhibition in cutaneous psoriasis distinguishes clinical response. *Sci Immunol* **9**, eadi2848 (2024).
